# Cholesterol synthesis enzyme SC4MOL is fine-tuned by sterols and targeted for degradation by the E3 ligase MARCHF6

**DOI:** 10.1016/j.jlr.2023.100362

**Published:** 2023-03-22

**Authors:** Lydia Qian, Nicola A. Scott, Isabelle M. Capell-Hattam, Eliza A. Draper, Nicole M. Fenton, Winnie Luu, Laura J. Sharpe, Andrew J. Brown

**Affiliations:** School of Biotechnology and Biomolecular Sciences, UNSW Sydney, Sydney, New South Wales, Australia

**Keywords:** posttranslational regulation, cholesterol biosynthesis, cholesterol metabolism, lipids, molecular biology, SC4MOL, MARCHF6, NSDHL, C4-demethylation, ubiquitin-proteasome system

## Abstract

Cholesterol biosynthesis is a highly regulated pathway, with over 20 enzymes controlled at the transcriptional and posttranslational levels. While some enzymes remain stable, increased sterol levels can trigger degradation of several synthesis enzymes via the ubiquitin-proteasome system. Of note, we previously identified four cholesterol synthesis enzymes as substrates for one E3 ubiquitin ligase, membrane-associated RING-CH-type finger 6 (MARCHF6). Whether MARCHF6 targets the cholesterol synthesis pathway at other points is unknown. In addition, the posttranslational regulation of many cholesterol synthesis enzymes, including the C4-demethylation complex (sterol-C4-methyl oxidase-like, SC4MOL; NAD(P)-dependent steroid dehydrogenase-like, NSDHL; hydroxysteroid 17-beta dehydrogenase, HSD17B7), is largely uncharacterized. Using cultured mammalian cell lines (human-derived and Chinese hamster ovary cells), we show SC4MOL, the first acting enzyme of C4-demethylation, is a MARCHF6 substrate and is rapidly turned over and sensitive to sterols. Sterol depletion stabilizes SC4MOL protein levels, while sterol excess downregulates both transcript and protein levels. Furthermore, we found *SC4MOL* depletion by siRNA results in a significant decrease in total cell cholesterol. Thus, our work indicates SC4MOL is the most regulated enzyme in the C4-demethylation complex. Our results further implicate MARCHF6 as a crucial posttranslational regulator of cholesterol synthesis, with this E3 ubiquitin ligase controlling levels of at least five enzymes of the pathway.

Cholesterol is an important lipid for mammals, where it is required for cell membranes, lipid rafts, and bile acid and steroid hormone production (1). Its excess is linked to cardiovascular disease (2), and some cancers (3), while too little impairs embryonic development (4). Thus, cholesterol synthesis must be tightly regulated. The formation of cholesterol is energetically expensive, requiring abundant oxygen and cofactors such as NADPH in more than 20 enzymatic reactions. The two rate-limiting enzymes are 3-hydroxy-3-methylglutaryl-CoA reductase (HMGCR) (5, 6), the target of the cholesterol-lowering drugs, statins, and squalene monooxygenase (SM) (7).

Flux is also controlled along the length of the pathway beyond the two established rate-limiting enzymes (8). This allows regulated accumulation of sterol intermediates, the importance of which is becoming increasingly appreciated. A growing list of studies implicate sterol intermediates in a range of biological functions from reversing cataracts (9) to suppressing protein aggregation and cytotoxicity in neurodegenerative disease states (10), to promoting oligodendrocyte formation (11), and even modulating immune function (12).

Post lanosterol, there are two parallel routes to cholesterol via the Bloch or Kandutsch-Russell pathways, where three methyl groups are removed from lanosterol to generate the 27-carbon frame of cholesterol. Lanosterol 14α demethylase (LDM) removes the methyl group at C14. Three enzymes then act sequentially in a functional complex to remove two methyl groups at C4, converting T-MAS (testis meiosis-activating sterol) to zymosterol (Fig. 1). First, SC4MOL (sterol-C4-methyl oxidase-like, also referred to by its gene name MSMO1) sequentially oxidizes one methyl group to produce a C4 carboxylic acid. Second, NSDHL (NAD(P)-dependent steroid dehydrogenase-like) catalyzes oxidative decarboxylation to yield a C3 keto-sterol product. Finally, HSD17B7 (hydroxysteroid 17-beta dehydrogenase) reduces the C3 ketone to produce the C4-demethylated sterol product. To remove both methyl groups from T-MAS for zymosterol production, two cycles of these reactions are required (13). The homologues in *Saccharomyces cerevisiae* (Erg25p, Erg26p, and Erg27p, respectively) are tethered together in the endoplasmic reticulum (ER) by a scaffolding protein, Erg28p (14), and human ERG28 may play an analogous role in cholesterol synthesis (15).

**Fig. 1:**
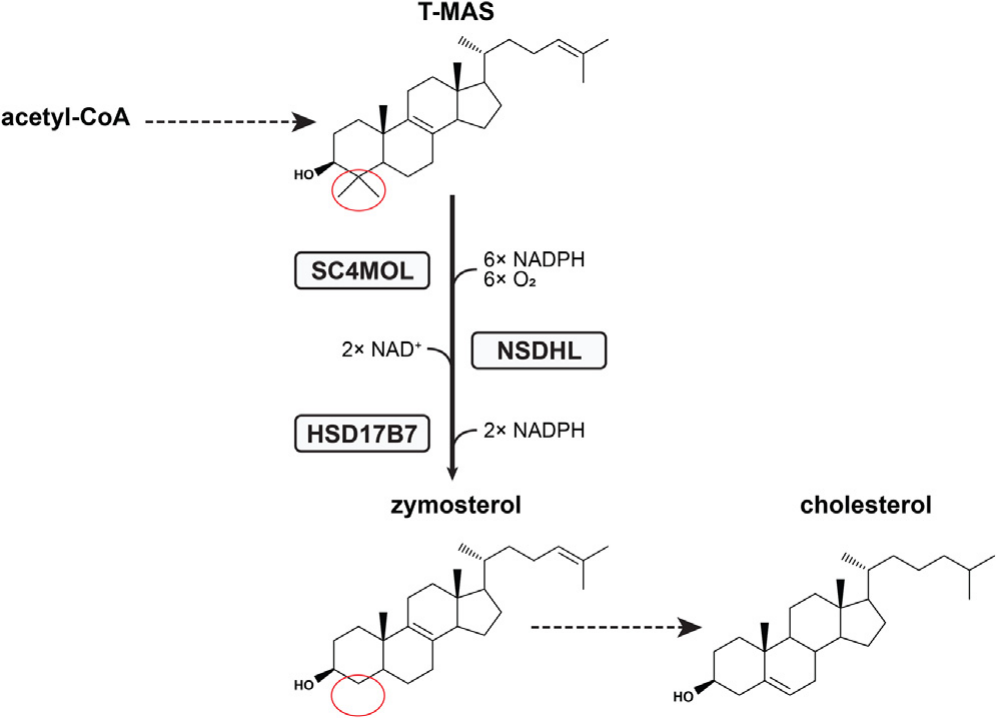
Conversion of T-MAS to zymosterol catalyzed by SC4MOL, NSDHL, and HSD17B7 in the Bloch pathway. Two cycles of this reaction are required. C4-demethylation is circled in red.

Cholesterol levels are rapidly regulated by posttranslationally controlling the levels of biosynthetic enzymes through ER-associated degradation (ERAD) and the ubiquitin-proteasome system. In this system, an E3 ligase ubiquitinates substrates for proteasomal degradation and thereby depletes enzyme levels. Previous work found the early, rate-limiting enzymes HMGCR and SM are rapidly turned over in response to sterols and degraded by the proteasome (5, 6, 7). HMGCR is targeted by several E3 ubiquitin ligases (16, 17), including the E3 ligase, membrane-associated RING-CH-type finger 6 (MARCHF6), although SM is its canonical substrate (18). Moreover, MARCHF6 is an important regulator of cholesterol biosynthesis as it targets two other synthesis enzymes, LDM and 24-dehydrocholesterol reductase (DHCR24). LDM acts midway in the pathway, controlling entry into either the Bloch or Kandutsch-Russell pathway. DHCR24 can funnel intermediates from the Bloch to the Kandutsch-Russell pathway, in addition to performing the final step in the Bloch pathway to produce cholesterol from desmosterol (19). However, not all cholesterol synthesis enzymes are targeted by MARCHF6 for degradation; we have found 7-dehydrocholesterol reductase (DHCR7), 14-dehydrocholesterol reductase (DHCR14), lanosterol synthase, and 3-β-hydroxysteroid-Δ^8^,Δ^7^-isomerase are not MARCHF6 substrates (19, 20, 21).

MARCHF6 likely helps to coordinate the control of lipid metabolism (22) as it targets many cholesterol synthesis enzymes and other lipid metabolism proteins including perilipin-2 (23), a lipid droplet-binding protein, mutant variants of a bile salt pump, BSEP (24), and the cholesterol transporter NPC1 (25). Other MARCHF6 substrates include RGS2 (26), a G protein signaling protein; DIO2 (27), the main thyroid hormone activator; and DHX9 (28), an RNA helicase involved in thyroid cancer (28), suggesting a role in signaling and cancer. *MARCHF6* mutations are linked with at least two neurological disorders. The chromosomal location of *MARCHF6* maps to the cri-du-chat critical region, and TTTA/TTTCA repeat expansions in the first *MARCHF6* intron are associated with familial adult myoclonic epilepsy (22, 29, 30). Recently, MARCHF6 was identified as an important regulator of ferroptosis, a form of cell death caused by iron-dependent lipid peroxidation modulated by the levels of the anabolic reductant NADPH (31). MARCHF6 promotes the degradation of ferroptosis effectors, p53, and acyl-CoA synthetase long-chain family member 4 (ACSL4). Moreover, NADPH binds to the evolutionarily conserved C terminus of MARCHF6, which is also involved in MARCHF6 substrate discrimination (31, 32). Consequently, MARCHF6 can act as an NADPH sensor to mediate cell death or survival (22, 31).

We hypothesized additional cholesterol synthesis enzymes are likely targeted by MARCHF6, in an elegant system evolved to rapidly shutdown the entire pathway at key steps. Here we sought to identify putative MARCHF6 substrates among the three enzymes comprising the C4-demethylation complex. In this study, using human and Chinese hamster ovary cell culture systems, we found SC4MOL is a MARCHF6 target, whereas NSDHL and HSD17B7 are not. We investigated the transcriptional and posttranslational regulation of SC4MOL and discovered it is rapidly turned over and sensitive to cell sterol levels. Furthermore, SC4MOL depletion results in a reduction in total cell cholesterol. Taken together, our findings support the concept that the initial enzyme in C4-demethylation, SC4MOL, is the most regulated step in this process.

## Materials and methods

### Cell culture

Chinese hamster ovary 7 (CHO-7) cells (kind gifts of Drs Brown and Goldstein, University of Texas Southwestern) were grown in DMEM/F12 medium supplemented with 5% (v/v) lipoprotein-deficient serum (prepared from newborn calf serum in-house) and containing penicillin (100 U/ml) and streptomycin (100 μg/ml). CHO-7 cell lines stably expressing SC4MOL-V5 or NSDHL-V5 were generated with CHO-7 FRT cells as described previously (33) and grown in medium containing 100 μg/ml Hygromycin B. Huh7 and HEK293T cells were grown in DMEM/high glucose supplemented with 10% (v/v) fetal calf serum and penicillin (100 U/ml) and streptomycin (100 μg/ml). For HEK293T cells, plates were coated with polyethylenimine before seeding to ensure increased adherence.

Cells were seeded in 6-well or 12-well plates, transfected with siRNA or plasmids the following day, and/or treated as described in the figure legends.

### Plasmids

The protein-coding sequences of *NSDHL*, *SC4MOL*, and *HSD17B7* were amplified from HeLaT cDNA and cloned into our in-house pcDNA5/FRT construct (33) with a C-terminal V5 tag, with a CMV promoter. NSDHL was then moved to a construct with a weaker TK promoter to reduce expression levels. The pcDNA3.1-DHCR24-V5, pcDNA3.1-V5-DHCR24 (34), and pcDNA3.1-SM-V5 (7) were previously generated. NanoBiT® plasmids were cloned following the protocol outlined in the NanoBiT® PPI Starter System (Promega). SC4MOL NanoBiT® constructs were previously generated (15). SM-V5 (7) was amplified by PCR and subcloned using SacI and NheI restriction enzyme cloning into plasmids supplied in the kit [pBiT1.1-C (TK/LgBiT), pBiT2.1-C (TK/SmBiT), pBiT1.1-N (TK/LgBiT), or pBiT2.1-N (TK/SmBiT)]. MARCHF6-V5 (35) constructs were made similarly, but with NheI and EcoRI restriction enzymes. All plasmids were confirmed via Sanger sequencing conducted by the Ramaciotti Centre for Genomics at UNSW Sydney. Primer sequences are provided in supplemental Table S1.

### siRNA transfections

Cells were seeded in 12-well plates and transfected with 25 nM siRNA [control (Sigma: SIC001-1NMOL), MARCHF6 (Sigma: SASI_Hs01_00105239), SC4MOL (Sigma: SASI_Hs01_00125914), NSDHL (Sigma: SASI_Hs01_00243652), ERG28 (SASI_Hs01_00143837)] using 2.5 μl Lipofectamine RNAiMAX (Life Technologies) for 24 h in media without antibiotics.

### Plasmid transfections

For plasmid transfections, HEK293T or Huh7 cells were seeded in 6-well or 12-well plates and transfected with 1 or 0.5 μg of plasmid, respectively, for 24 h, using Lipofectamine 3,000 as per manufacturer’s instructions.

### Treatments

Pretreatment and treatment media were DMEM/F12 media supplemented with 5% (v/v) lipoprotein-deficient serum, penicillin (100 U/ml) and streptomycin (100 μg/ml) and statin (compactin, 5 μM) and mevalonate (50 μM) to deplete cellular cholesterol.

For the Amplex Red assay, after siRNA transfection, Huh7 cells were washed once with PBS and refreshed with DMEM/high glucose supplemented with 10% (v/v) lipoprotein-deprived serum prepared in-house from fetal calf serum.

Cycloheximide (Sigma), MG132 (Sigma), and compactin (Sigma) were dissolved in DMSO. Mevalonate (Sigma) was dissolved in ethanol. Cholesterol-cyclodextrin (Sigma) was dissolved in filtered MilliQ water. Appropriate vehicle controls were used. Concentration of drugs used and treatment time are indicated in the figure legends.

### Quantitative real-time PCR

Cells were seeded in triplicate wells and total RNA was extracted in Tri-Reagent. Superscript III reverse transcriptase (Invitrogen) was used for cDNA synthesis, following manufacturer’s instructions. Quantitative real-time PCR (qRT-PCR) was conducted using the QuantiNova Real-Time PCR kit (Qiagen), and gene expression levels of *MARCHF6*, *SC4MOL*, *NSDHL*, *HSD17B7*, and *ERG28* were normalized to the housekeeping gene, *porphobilinogen deaminase* (*PBGD*), using the ΔΔCT method. mRNA levels of interest were normalized to the housekeeping gene *PBGD* and then normalized to the control, set to 1. Primer sequences are listed in supplemental Table S1.

### Western blotting

Cells were lysed in RIPA buffer [20 mM Tris-HCl (pH 7.4), 0.1% (w/v) SDS, 1% (w/v) IGEPAL CA-630, 0.5% (w/v) sodium deoxycholate, 150 mM NaCl, 5 mM EDTA, 1 mM sodium orthovanadate] supplemented with 2% (v/v) protease inhibitor cocktail (Sigma-Aldrich) and passed through a 23-gauge needle 20 times, rotated at 4°C for 30 min, and then centrifuged at 20,000 *g* at 4°C for 15 min. Protein concentration was measured using a Pierce Bicinchoninic Acid Protein Kit (Thermo Fisher Scientific) according to manufacturer’s instructions and normalized in lysis buffer with Laemmli buffer. Proteins were resolved by 10% (w/v) SDS-PAGE and transferred to nitrocellulose membranes (Bio-Rad). Membranes were blocked in 5% (w/v) skim milk in PBS with 1% Tween-20 (PBST) and incubated with the following primary antibodies: mouse anti-V5 tag (1:5,000; Life Technologies; R960-25), rabbit anti-SM (1:5,000; ProteinTech; 12544-1-AP), rabbit anti-vinculin (1:2,000; Abcam; ab129003), mouse anti-α-tubulin (1:10,000; Sigma-Aldrich; T5168). Following primary antibody incubation, membranes were incubated with HRP donkey anti-rabbit (1:10,000; Jackson ImmunoResearch Laboratories; 711-035-152) or donkey anti-mouse secondary antibodies (1:10,000 Jackson ImmunoResearch Laboratories; 715-035-150) for 1 h and imaged using the ImageQuant LAS 500 (GE Healthcare). All primary antibodies were diluted in 5% (w/v) bovine serum albumin in PBST and 0.02% (w/v) sodium azide. All secondary antibodies were diluted 1:10,000 in 5% (w/v) skim milk in PBST. Blots were washed with PBST for 3 × 10 min between blocking and antibody incubations. Full immunoblots for V5, vinculin, endogenous SM, and α-tubulin are shown in supplemental Fig. S1.

### NanoBiT® assay

For NanoBiT® transfections, HEK293T cells in 12-well plates were cotransfected for 24 h with a total of 1,050 ng of plasmids, using Lipofectamine LTX as per manufacturer’s instructions. For MARCHF6 and SM constructs, 350 ng of plasmid containing the LgBiT or SmBiT tagged reporter construct was used, and for the lowly expressing SC4MOL, 700 ng of plasmid containing the LgBiT or SmBiT tagged reporter was used. Plasmid amounts were kept constant between conditions by addition of an empty vector. Corresponding HaloTag negative control plasmids were also used as per manufacturer’s instructions. After transfection, cells were split into a white opaque 96-well plate and left for 8 h to adhere.

Cells were then treated with 5 μM MG132 for 16 h. The medium was replaced with 50 μl serum-free Opti-MEM and incubated for 30 min at 37°C. A volume of 50 μl reagent mastermix (1 μl of Nano-Glo® Live Cell Substrate, 19 μl of Nano-Glo® LCS Dilution Buffer, and 30 μl of Opti-MEM per well) was added to each well. Luminescence was measured in a CLARIOstar microplate reader at 37°C, as per Promega’s instructions. Raw luciferase activity was normalized to the appropriate LgBiT:HaloTag negative control pair.

### Membrane isolation and protease protection assay

CHO-7 cells were cotransfected with 4 μg SC4MOL-V5, and 1 μg of V5-DHCR24 or DHCR24-V5 (34) in a 10-cm dish for 24 h. To prepare membranes for protease protection assays, cells were washed and scraped in PBS and centrifuged at 1,000 *g* for 5 min at 4°C. The pellet was resuspended in Buffer A [10 mM Hepes-KOH (pH 7.4), 10 mM KCl, 1.5 mM MgCl_2_, 5 mM sodium EDTA, 5 mM sodium EGTA, 250 mM sucrose] and passed through an 18-gauge needle 50 times. The cell lysate was centrifuged at 1,000 *g* for 5 min at 4°C. The supernatant was further centrifuged at 20,000 *g* for 15 min at 4°C. The resulting pellet (membrane fraction) was resuspended in 65 μl Buffer A.

Resuspended membranes were treated with 0, 0.02, 0.2, or 2 μg of trypsin in the presence or absence of 0.25% Triton X in Buffer A, for 30 min at 30°C. Loading buffer and heat inactivation at 95°C for 10 min stopped the reaction. Proteins were separated by SDS-PAGE and visualized by Western blotting.

### Site-directed mutagenesis

The pcDNA5/FRT SC4MOL-V5 plasmid was used for megaprimer site-directed mutagenesis (36) to generate the K to R mutant plasmids. Primer sequences are listed in supplemental Table S1.

### Amplex Red cholesterol assay

Cell cholesterol levels were measured using an Amplex™ Red Cholesterol Assay Kit (Thermo Fisher Scientific). Cells were washed three times with PBS, and 250 μl modified RIPA [50 mM Tris-HCl (pH 8.0), 150 mM NaCl, 0.1% (w/v) SDS, 1.5% (w/v) IGEPAL CA-630, 0.5% (w/v) sodium deoxycholate, 2 mM MgCl_2_] supplemented with 2% (v/v) protease inhibitor cocktail (Sigma-Aldrich) was added to the wells. Cells were lysed by freeze-thawing from −80°C to room temperature. Lysates were rotated for 15 min at 4°C, and cellular debris was pelleted at 17,000 *g* for 15 min. Cell lysates were diluted 1:8 with modified RIPA. Amplex Red assay was conducted as per manufacturer’s instructions. Protein concentration was measured using a Pierce Bicinchoninic Acid Protein Kit (Thermo Fisher Scientific) according to manufacturer’s instructions. Total cholesterol content was normalized to protein content for each sample. Relative cholesterol content after siRNA knockdown was calculated by normalizing to the control, set to 1.

### Bioinformatic analysis

Multiple sequence alignment of SC4MOL protein sequence from human (UniProt: Q15800), macaque (Q4R4Q4), mouse (Q9CRA4), rat (O35532), pig (Q6UGB2), chicken (Q5ZLL6), zebrafish (Q7ZW77), and yeast Erg25 (P53045) was conducted in Geneious v11.0.4 with default settings. The complete human amino acid sequence of SC4MOL (Q15800) was used to predict membrane topology with TOPCONS (37).

### Data presentation and statistical analysis

For Western blot analysis, densitometry was conducted and normalized to the control in the first lane, set to 1.

To account for changes in basal protein expression in transient transfections and *MARCHF6* knockdowns, we calculated MARCHF6 response of SC4MOL with respect to each transfected plasmid or siRNA condition, as described in previous work (38). Densitometry was first conducted, followed by normalization of the fold-increase for each construct after *MARCHF6* siRNA knockdown to the fold-increase for WT, which was set to 1. A value close to 1 indicates the MARCHF6 response is similar to that of WT.

Images and densitometry were analyzed in LI-COR Image Studio Lite (Version 5.2). Graphs and statistical analyses were made with GraphPad Prism (Version 9.3.1).

## Results

### SC4MOL is a likely MARCHF6 target

We previously identified four cholesterol biosynthesis enzymes targeted by MARCHF6 (18, 22). Here we used a similar siRNA knockdown approach to identify if MARCHF6 targets any enzymes of the C4-demethylation complex, SC4MOL, NSDHL, and HSD17B7.

**Fig. 2:**
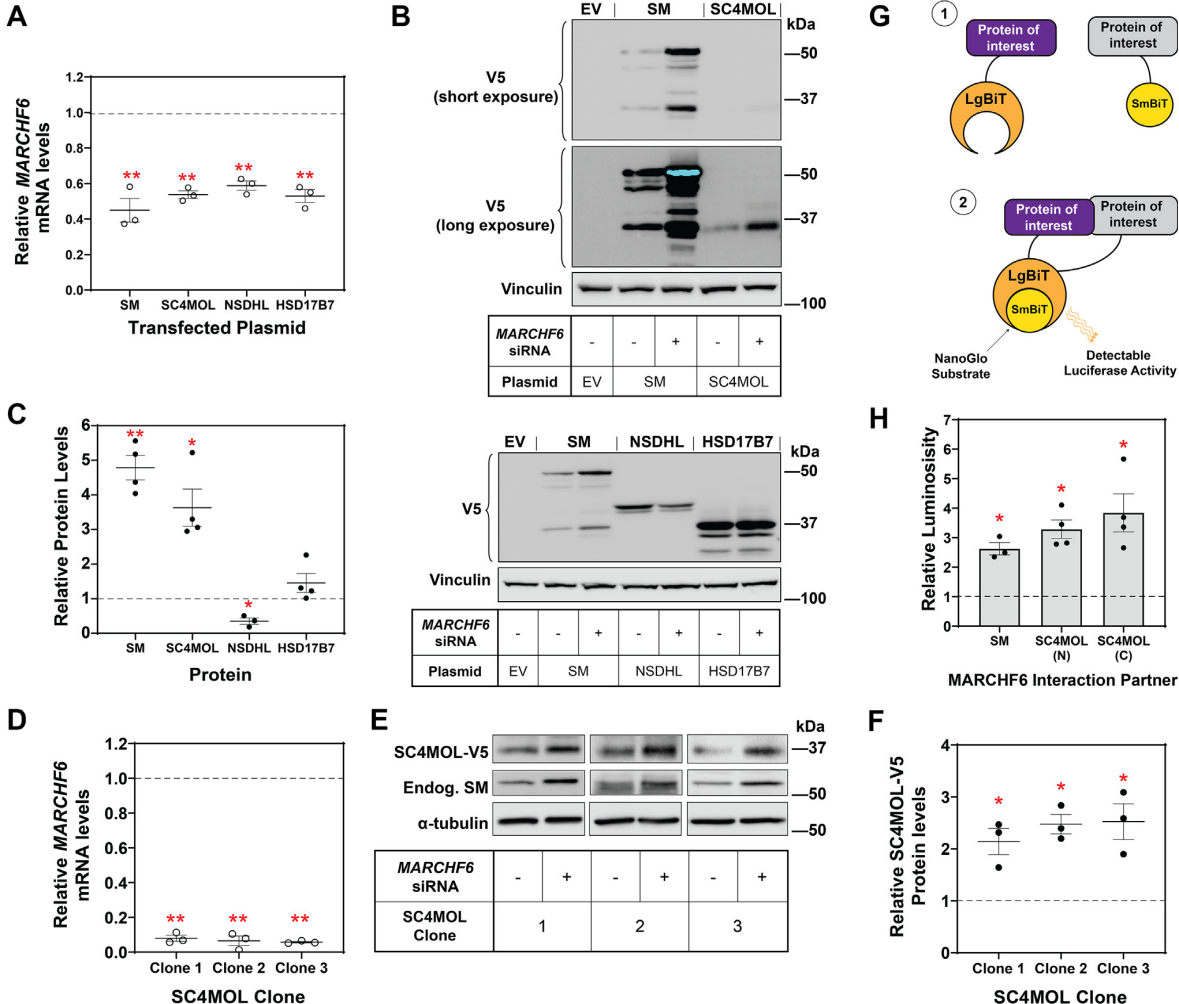
SC4MOL is a likely MARCHF6 substrate. A–C, HEK293T cells were transfected with 25 nM control (−) or *MARCHF6* (+) siRNA for 24 h, followed by transfection with the indicated plasmid for 24 h. A: *MARCHF6* levels were measured using qRT-PCR and normalized to the housekeeping gene *PBGD* and control condition, which was set to 1 (represented by dashed line). B: Protein levels were measured by Western blotting with V5 and endogenous vinculin antibodies. C: Levels of each transfected protein after *MARCHF6* siRNA knockdown were normalized to the control knockdown, set to 1 (represented by dashed line). D–F: Three clones of CHO-SC4MOL-V5 cells were transfected with 25 nM control (−) or *MARCHF6* (+) siRNA for 24 h. D: *MARCHF6* mRNA levels were measured using qRT-PCR and normalized to the housekeeping gene *PBGD* and to the control condition, which was set to 1 (represented by dashed line). E: Protein levels were analyzed by Western blotting with V5, endogenous SM, and α-tubulin antibodies. F: Relative protein levels were measured using ImageStudio Lite and normalized to the control condition, which was set to 1 (represented by dashed line). G: Schematic of NanoBiT® luciferase system for protein-protein interaction. *1*) Proteins of interest (MARCHF6 and its interacting partners, SM or SC4MOL) are fused with LgBiT™ or SmBiT™ constructs. *2*) If the proteins of interest interact with MARCHF6, a functional Nano Luciferase is formed, which acts on the NanoGlo substrate to produce luminescence. H: Relative luminosity is normalized to the response of the appropriate LgBiT:HaloTag negative control pair, set to 1 (represented by the dashed line). Data presented as mean ± SEM from n = 3 independent experiments performed in triplicates (A, D) or n = 3–4 experiments (C) or n = 3 experiments (F) or n = 3–4 independent experiments performed in duplicates (H), where ∗ *P* < 0.05, ∗∗*P* < 0.01. A paired Student’s two-tailed *t* test was conducted between control and *MARCHF6* siRNA samples for each protein (A, C, D, and F) or the appropriate negative control and MARCHF6-interactor pair (H).

As an initial screen, we used siRNA to deplete *MARCHF6* mRNA (Fig. 2A–C), then expressed our proteins of interest fused to a V5 epitope tag. The canonical substrate SM (18) was used as a positive control. We found an increase in SC4MOL levels after *MARCHF6* knockdown, indicating that it is a putative MARCHF6 target (Fig. 2B, C). NSDHL and HSD17B7 levels did not increase, suggesting they are unlikely to be MARCHF6 substrates. Unexpectedly, NSDHL levels decreased after *MARCHF6* knockdown. Thus, we generated stable CHO-7 cell lines expressing SC4MOL-V5 or NSDHL-V5 as a more robust and physiological system for detecting potential MARCHF6 substrates, as these express a single copy of the introduced gene per cell. In these cells, *MARCHF6* was knocked down by >90% using siRNA (Fig. 2D, supplemental Fig. S2A), which resulted in a 2- to 3-fold increase in SC4MOL (Fig. 2E, F). We observed this result in seven independent clones (three clones shown in Fig. 2), providing further evidence MARCHF6 is involved in its turnover. By contrast, NSDHL-V5 levels did not change after *MARCHF6* knockdown, suggesting NSDHL is not a MARCHF6 substrate (supplemental Fig. S2B, C).

To further explore the interactions between MARCHF6 and SC4MOL, we used NanoLuc® Binary Technology (NanoBiT). This is a complementation assay where two proteins of interest are fused to complementary parts of a luciferase molecule, and if the proteins interact, functional luciferase is produced and luminescence can be measured (Fig. 2G). In our experience, we consistently observe low ectopic levels of both SC4MOL (Fig. 2A) and MARCHF6 (35). To improve the expression of SC4MOL and MARCHF6 NanoBiT constructs, and consequently luminescence signal, we treated cells with a proteasome inhibitor and successfully increased their levels (supplemental Fig. S3). As a positive control, we confirmed MARCHF6 and its canonical substrate SM interact in this system (Fig. 2H). Luminescence was produced by MARCHF6 and SC4MOL, regardless of whether the fusion was to the N or C terminus of the protein, consistent with MARCHF6 and SC4MOL interacting (Fig. 2H). Together with the siRNA data, these findings indicate that MARCHF6 targets SC4MOL.

### SC4MOL is degraded by the proteasome and its C-terminal lysines are not targeted by MARCHF6

**Fig. 3:**
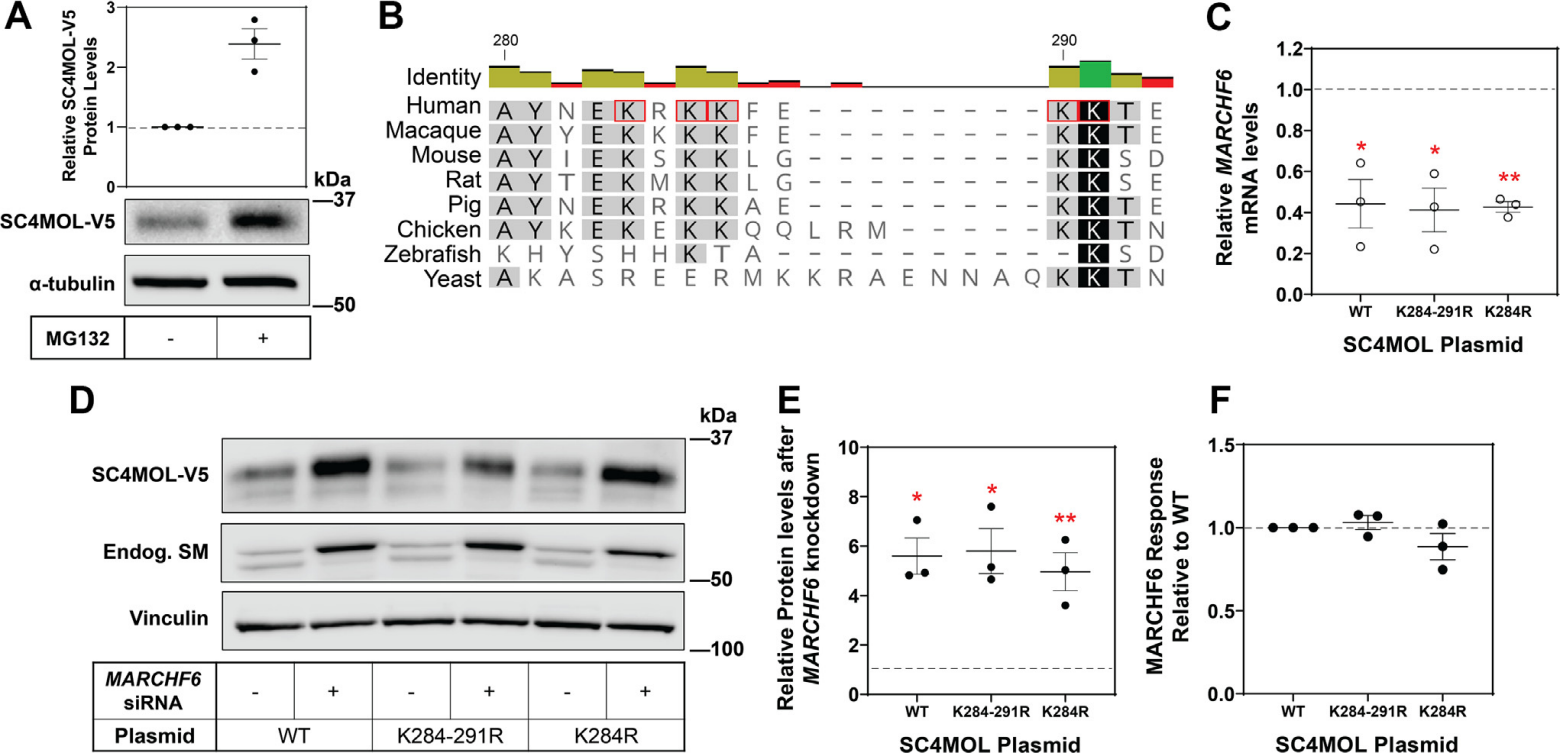
SC4MOL is degraded by the proteasome, and MARCHF6 does not target lysines at SC4MOL’s C terminus. A: CHO-SC4MOL-V5 cells were treated with or without 10 μM MG132 for 8 h. B: Multiple sequence alignment of SC4MOL protein sequences from human, vertebrates, and yeast Erg25, conducted in Geneious v11.0.4. Residues with 100%, 99%–60% or <60% identity highlighted in black, gray, and white, respectively. Potential ubiquitinated lysines selected for site-directed mutagenesis are boxed in red. C–F: HEK293T cells were transfected with 25 nM control or *MARCHF6* siRNA for 24 h, followed by transfection with wild type (WT), K284-291R or K284R SC4MOL mutant plasmids for 24 h. C: mRNA levels of *MARCHF6* were measured using qRT-PCR and normalized to the housekeeping gene *PBGD* and control siRNA condition, which was set to 1 for each plasmid (represented by dashed line). D: Protein levels were analyzed by Western blotting with V5 (SC4MOL), endogenous SM and vinculin antibodies. E: Relative protein levels were measured using ImageStudio Lite and normalized to the control siRNA condition for each, which was set to 1 (represented by the dashed line). F: The fold increase for each SC4MOL construct after *MARCHF6* siRNA knockdown was normalized to the fold increase for WT, which was set to 1 (represented by the dashed line). Data presented as mean ± SEM from n = 3 independent experiments each performed in triplicate (C) or from n = 3 independent experiments (E, F) where ∗*P* < 0.05, ∗∗*P* < 0.01. A paired Student’s two-tailed *t* test was conducted between control and *MARCHF6* siRNA samples for wild-type and mutant samples.

To determine if SC4MOL is turned over by the ubiquitin-proteasome system, we treated our SC4MOL-V5 stable cells with a proteasome inhibitor, MG132. SC4MOL accumulated by 2- to 3-fold after proteasome inhibition (Fig. 3A), bolstering our observation that SC4MOL NanoBiT constructs were also stabilized upon MG132 addition (supplemental Fig. S3). Together, these data indicate SC4MOL is degraded by the proteasome.

PhosphoSitePlus (39) was examined to determine potential MARCHF6 ubiquitination sites on SC4MOL. K284 was the most likely candidate, with a total of 32 ubiquitination references recorded, including eight publications. PhosphositePlus also indicated K284 ubiquitination is conserved in mice, and a recent paper showed regulation in human SC4MOL at the K284 ubiquitination site by arsenite (40). K284 occurs in a lysine-rich stretch on the C terminus with proximal lysines at 286, 287, 290, and 291, although of these residues only K284 has been reported to be ubiquitinated (39). However, it is possible MARCHF6 may also ubiquitinate one or more of these adjacent lysines. Of note, K291 is conserved across all species examined, with the other lysines conserved in most vertebrate species (Fig. 3B).

To assist in our identification of SC4MOL’s ubiquitination sites, we considered whether these C-terminal lysines are exposed in the cytosol and therefore potentially positioned to be recognized by MARCHF6’s RING domain. The yeast homologue of MARCHF6, Doa10, recognizes cytosolic-facing regions of proteins (41), and MARCHF6 mediates proteasomal degradation of a soluble reporter protein at the cytosolic face of the ER membrane (42). We first examined the partial topology of SC4MOL. *In silico* predictions by TOPCONS (37) indicated conflicting topologies; four prediction programs suggested an ER luminal topology for the C terminus, while two programs predicted a cytosolic topology (supplemental Fig. S4A). To experimentally determine the orientation of the C terminus of SC4MOL, we conducted a protease protection assay with a C-terminal V5 epitope tag on SC4MOL expressed in HEK293T cells. Cytosolic portions of the protein are accessible to trypsin cleavage, while portions of the protein buried in the ER are not. We previously used this method to successfully map the topology of another cholesterogenic enzyme, DHCR24 (34), and therefore, DHCR24 constructs with V5 at the N terminus or C terminus were cotransfected with SC4MOL-V5. As published previously, DHCR24 results indicate a luminal N terminus and cytosolic C terminus. To ensure no DHCR24 trypsinized fragments are produced at the size of SC4MOL, a protease protection assay was conducted with DHCR24 constructs alone (supplemental Fig. S4B, C). Similar to DHCR24-V5, SC4MOL-V5 was digested by trypsin, indicating that the C terminus is trypsin accessible and therefore exposed in the cytosol and accessible to the MARCHF6 RING domain (supplemental Fig. S4D, E).

We used site-directed mutagenesis to make conservative lysine-to-arginine mutations at K284 and at all lysines between residues 284 and 291 (Fig. 3B). Although MARCHF6 was successfully knocked down in all cells (Fig. 3C), we found no blunting of the MARCHF6 response (Fig. 3D–F) for either mutant, suggesting MARCHF6 does not require these five C-terminal lysines of SC4MOL to promote its degradation.

### SC4MOL is relatively labile

**Fig. 4:**
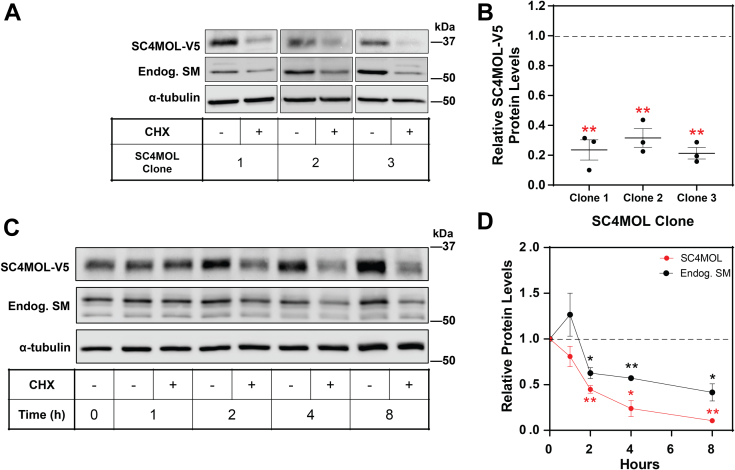
SC4MOL is rapidly turned over. A, B: Three clones of CHO-SC4MOL-V5 were treated with or without 10 μg/ml cycloheximide (CHX) for 8 h. A: Protein levels were analyzed by Western blotting with V5, endogenous SM, and α-tubulin antibodies. B: Protein levels relative to the control condition for each clone, set to 1 (represented by dashed line). A Student’s paired two-tailed *t* test was conducted between the control and CHX (+) condition. C, D: CHO-SC4MOL-V5 cells were pretreated with 5 μM compactin and 50 μM mevalonate for 16 h and then treated with 10 μg/ml CHX for 0–8 h. C: Protein levels were analyzed by Western blotting with V5, endogenous SM, and α-tubulin antibodies. D: Protein levels are relative to the paired control for each time point, set to 1 for each protein (represented by dashed line). A paired Student’s two-tailed *t* test was conducted between CHX(−) and CHX(+) for each time point for SC4MOL-V5 and endogenous SM. B, D: All data are presented as mean ± SEM from n = 3 independent experiments each, where ∗*P* < 0.05, ∗∗*P* < 0.01.

We next examined the stability of SC4MOL protein by treating our stably expressing cells with the protein synthesis inhibitor cycloheximide for 8 h in medium containing statin and mevalonate. SC4MOL-V5 underwent significant degradation with less than 40% of the protein remaining for each of three clones (Fig. 4A, B). As clone 3 of SC4MOL had the greatest response to cycloheximide and *MARCHF6* siRNA knockdown (Figs. 2E and 4A), it was selected for subsequent experiments.

As SC4MOL was highly turned over after 8 h, we performed a cycloheximide time course to measure its turnover rate. We found SC4MOL is rapidly turned over, with less than 50% remaining after 2 h of cycloheximide treatment (Fig. 4C, D). SC4MOL levels continued to decrease throughout the time course, with only ∼10% of protein remaining after 8 h of protein synthesis inhibition (Fig. 4C, D). Notably, NSDHL showed no turnover after 8 h (supplementary Fig. S5). Curiously, cholesterol depletion by statin treatment markedly increased SC4MOL levels over 8 h (Fig. 4C). This may indicate SC4MOL’s turnover is sensitive to cell sterol status.

### SC4MOL mRNA and protein are sensitive to cell sterol status

Considering statin treatment increased SC4MOL protein levels (Fig. 4C), we next investigated whether SC4MOL is regulated in a similar manner to other cholesterol synthesis enzymes. SM, DHCR14, and DHCR7 (7, 20, 21) are degraded in the presence of cholesterol, while HMGCR’s degradation is triggered by oxysterols and other sterol intermediates (43).

**Fig. 5:**
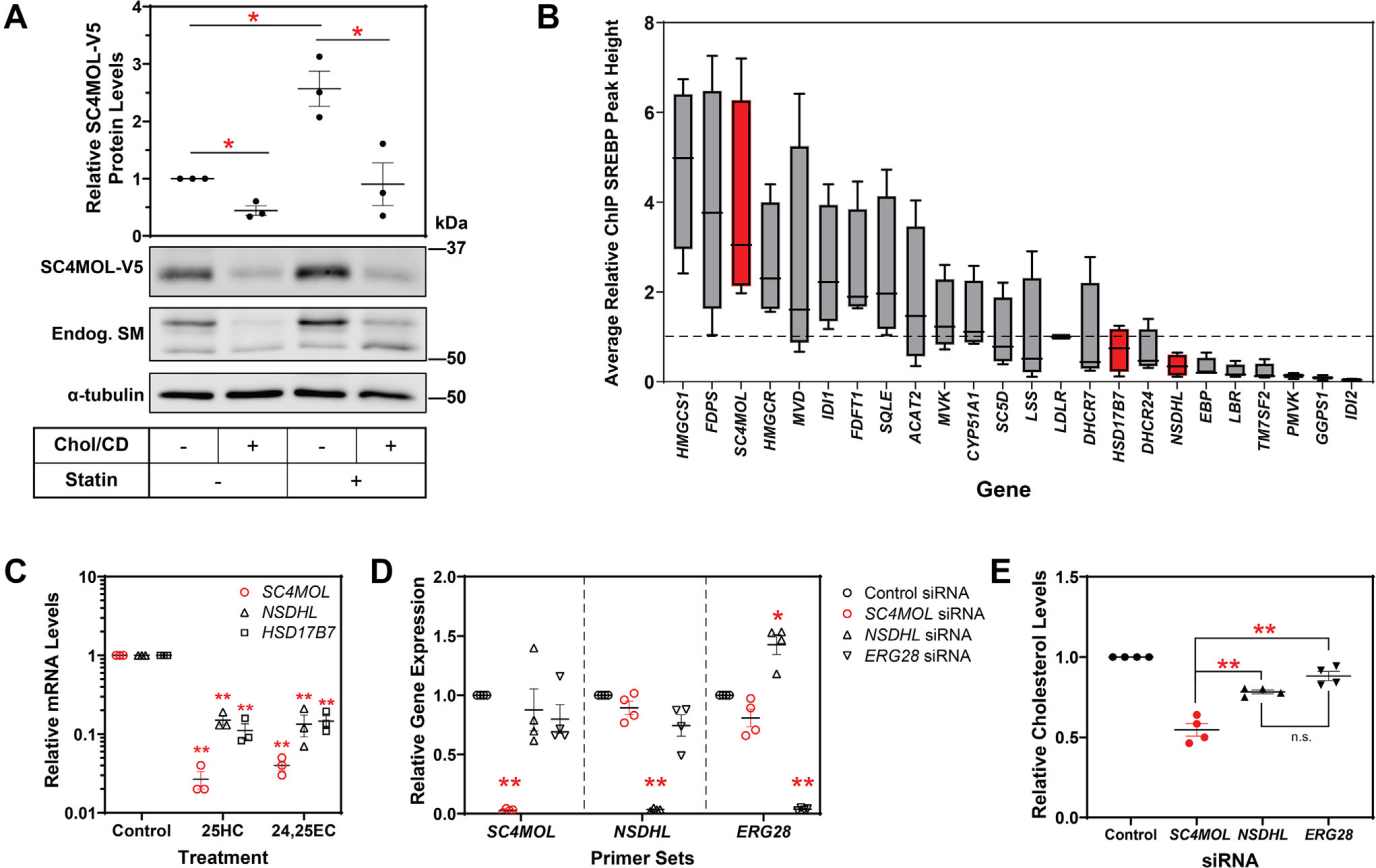
Sterols regulate SC4MOL transcriptionally and posttranslationally. A: CHO-SC4MOL-V5 cells were pretreated with or without media containing 5 μM compactin and 50 μM mevalonate for 16 h and treated with 20 μg/ml cholesterol/cyclodextrin (Chol/CD) for 8 h. Protein levels were analyzed by Western blotting with V5, endogenous SM, and α-tubulin antibodies. B: The maximum peak heights from four publicly available SREBP ChIP-seq databases on UCSC Genome browser (44, 45) were extracted (GM78 SREBP-2, HepG2 + insulin SREBP-1, HepG2 + pravastatin SREBP-1, and HepG2 + pravastatin SREBP-2). These were normalized to the peak height of *LDLR* in each set (represented by the dashed line) and then normalized peak heights were averaged across all four sets. *SC4MOL*, *HSD17B7*, and *NSDHL* are indicated in red. C: Previously generated sterol depleted cDNA sets from Huh7 cells treated for 24 h with 10 μM 25-hydroxycholesterol (25HC) or 10 μM 24(*S*),25-epoxycholesterol (24,25EC) (21). Levels of *SC4MOL*, *NSDHL*, and *HSD17B7* mRNA were measured using qRT-PCR and normalized to *PBGD*. mRNA levels are relative to the control condition, which was set to 1. A Student’s two-tailed paired *t* test was conducted between the control and oxysterol treated conditions. D: Huh7 cells were transfected for 24 h with 25 nM indicated siRNA, then treated for 24 h in sterol-depleted media. Total RNA was harvested, and levels of *SC4MOL*, *NSDHL*, and *ERG28* were measured using qRT-PCR and normalized to *PBGD*. mRNA levels are relative to the control siRNA, which was set to 1. A Student’s two-tailed paired *t* test was conducted between the control and siRNA for each gene. E: Cells were transfected and treated as in (D). Cholesterol content of cells was measured using an Amplex Red assay kit and normalized to protein content. A Student’s two-tailed paired *t* test was conducted between pairs of *SC4MOL* siRNA, *NSDHL* siRNA, and *ERG28* siRNA. Data are presented as mean ± SEM of n = 3 independent experiments (A, C) or n = 4 independent experiments (D, E). Experiments from (C–E) were performed in triplicate, where n.s. = nonsignificant, ∗*P* < 0.05, ∗∗*P* < 0.01.

Cells were pretreated with statin to decrease cell cholesterol levels and treated with cholesterol solubilized in methyl-β-cyclodextrin to increase cholesterol levels. Depleting cell cholesterol by statin treatment more than doubled SC4MOL-V5 levels after 8 h, suggesting the protein is very sensitive to cell cholesterol status (Fig. 5A). Treatment with cholesterol resulted in the degradation of SC4MOL after 8 h, with this decrease in protein levels seen under both conditions of normal cholesterol and cholesterol depletion (Fig. 5A). These findings indicate a high cholesterol status is a cue for SC4MOL protein turnover.

We next considered if *SC4MOL*, *NSDHL*, and *HSD17B7* are transcriptionally responsive to sterols. Although all three genes are transcriptionally responsive to sterols, *SC4MOL* is likely to be the most sensitive, based on chromatin immunoprecipitation (ChIP)-seq data (44, 45) (Fig. 5B) and our experiments where the transcripts were measured by qRT-PCR after oxysterol treatment (Fig. 5C). Oxysterol treatment significantly downregulated transcripts of all C4-demethylation genes, with the greatest response seen in *SC4MOL* (Fig. 5C). Indeed, *SC4MOL* was significantly lower than *NSDHL* and *HSD17B7* after 25-hydroxycholesterol treatment. SC4MOL is therefore highly responsive to sterols both transcriptionally and posttranslationally.

### Depletion of SC4MOL reduces total cell cholesterol levels

SC4MOL is the first enzyme to catalyze the C4-demethylation reaction (Fig. 1), degraded by MARCHF6 (Fig. 2) and sensitive to sterols (Fig. 5A–C), suggesting it may be the most regulated enzyme of the C4-demethylation step.

We used siRNA knockdown in the human liver cell line, Huh7, to deplete transcript levels of *SC4MOL*, *NSDHL*, or *ERG28*, implicated in the C4-demethylation complex (15) and measured total cell cholesterol via an Amplex Red assay. After successful knockdown of all transcripts (Fig. 5D), cholesterol levels were significantly decreased compared with the control (Fig. 5E). However, *SC4MOL* siRNA knockdown led to the most striking depletion, with cholesterol levels after *SC4MOL* knockdown significantly lower than both the *NSDHL* knockdown and the *ERG28* knockdown. Cholesterol levels after *NSDHL* knockdown or *ERG28* knockdown were not significantly different from each other. This finding suggests of these enzymes, SC4MOL is an important contributor to total cell cholesterol, providing further evidence for SC4MOL as the most regulated enzyme of C4-demethylation in cholesterol synthesis.

## Discussion

Cholesterol synthesis is controlled at multiple steps in the pathway by the E3 ubiquitin ligase MARCHF6 (22). In this study, we screened for MARCHF6 targets among the three mammalian C4-demethylation enzymes. Of these, we identified SC4MOL as the only MARCHF6 substrate (Fig. 2 and supplemental Fig. S2), with an interaction detected between this E3 ligase and SC4MOL using a split-luciferase reporter assay (Fig. 2H). We also found SC4MOL is degraded by the proteasome (Fig. 3A and supplemental Fig. S3), readily turned over under basal conditions (Fig. 4), and is sensitive to cell sterol status (Fig. 5). *SC4MOL* is also highly responsive to sterols transcriptionally (Fig. 5B, C). Low cholesterol stabilizes SC4MOL protein levels, while high cholesterol is a trigger for its degradation (Fig. 5A). Depletion of *SC4MOL* by siRNA markedly decreases total cell cholesterol levels (Fig. 5D, E).

To investigate whether endogenous SC4MOL behaves in a similar manner to ectopic protein, we tested two endogenous antibodies according to the manufacturers’ instructions. Although many bands were seen, none corresponded to SC4MOL (supplemental Fig. S6). Hence, translation of our findings to endogenous SC4MOL awaits further investigation.

Taken together, SC4MOL appears to be the most regulated component of the C4-demethylation complex. We routinely observed much lower ectopic levels of SC4MOL protein when compared with NSDHL and HSD17B7 (Fig. 2A), consistent with its labile nature. In addition, SC4MOL is differentially regulated to NSDHL and HSD17B7. From our initial screen, it appears to be the only C4-demethylation enzyme targeted by MARCHF6 (Fig. 2 and supplemental Fig. S2), displaying a rapid basal turnover rate (Fig. 4). By contrast, NSDHL levels are stable (supplemental Fig. S5). Of the three C4-demethylation enzymes, it seems intuitive for the first acting enzyme, SC4MOL, to be the most regulated. We propose MARCHF6 can control flux through the C4-demethylation step by modulating SC4MOL protein levels, while NSDHL and HSD17B7 remain constitutively expressed. Nevertheless, further characterization of the regulation of NSDHL and HSD17B7 is warranted.

In yeast, the SC4MOL homologue, Erg25p, is 38% identical to the human isoform and is an ERAD target (46). Turnover of Erg25p can be rescued by the double knockout of the principal yeast E3 ubiquitin ligases involved in ERAD, Hrd1 and Doa10, but not knockout of either individually (46). Considering Doa10 is the yeast homologue of MARCHF6, posttranslational regulation of this C4-demethylation enzyme may be conserved to some extent, although mammals may possess further specialization since we found knockdown of *MARCHF6* alone was sufficient to stabilize human SC4MOL.

SC4MOL regulation is one facet of the complex and coordinated system for cell cholesterol control. Although cholesterol is the trigger for posttranslational degradation of some cholesterol biosynthesis enzymes (SM (7), DHCR14 (21), and DHCR7 (20)), sterol intermediates can also serve as triggers. Indeed, C4-dimethyl sterols, including SC4MOL’s substrate T-MAS, prompt turnover of the first rate-limiting enzyme, HMGCR (43), and the enzyme preceding SC4MOL, DHCR14 (21). Moreover, these sterol intermediates, including T-MAS, can suppress sterol regulatory element-binding protein 2 (SREPB2) cleavage and thus transcriptionally downregulate cholesterol biosynthesis genes (43). By fine-tuning SC4MOL levels, T-MAS accumulation can feedback transcriptionally and posttranslationally on the preceding portion of the pathway. It is very likely that the products of C4 demethylation and other sterol intermediates can also feedback upon SC4MOL to regulate its levels. Our previous work on the cholesterol synthesis enzymes DHCR14 and DHCR7 indicated many intermediates, such as T-MAS and zymosterol, can trigger their degradation (20, 21). Further investigation of the sensitivity of SC4MOL degradation to a panel of sterols is warranted.

SC4MOL’s substrate T-MAS appears to have tissue-specific function. T-MAS is detected in very low quantities in primary human hepatocytes (0.01% of total sterols) (47), suggesting it primarily acts as a cholesterol biosynthesis intermediate in the liver. However, the testes contains a higher proportion of T-MAS, where in the mouse it represents 4% of sterols (48). T-MAS and another C4-dimethyl-sterol, follicular fluid-meiosis activating sterol, were initially implicated in meiosis activation in vitro, although their current role remains controversial. Nevertheless, their enrichment in the gonads suggests tissue-specific action and an in vivo role of T-MAS and follicular fluid-meiosis activating sterol in germ cell development has been hypothesized (49).

Beyond reproduction, C4-dimethyl sterols have other potent biological activities. Cell signaling and maturation are other processes requiring sterol intermediates. SC4MOL is involved in epidermal growth factor receptor signaling, a pathway upregulated in cancer. SC4MOL knockdown sensitizes tumor cells to epidermal growth factor receptor-inhibiting chemotherapeutics (50). Moreover, the accumulation of these sterol intermediates or the suppression of SC4MOL via genetic or pharmacological means drives enhanced oligodendrocyte formation (51).

**Fig. 6:**
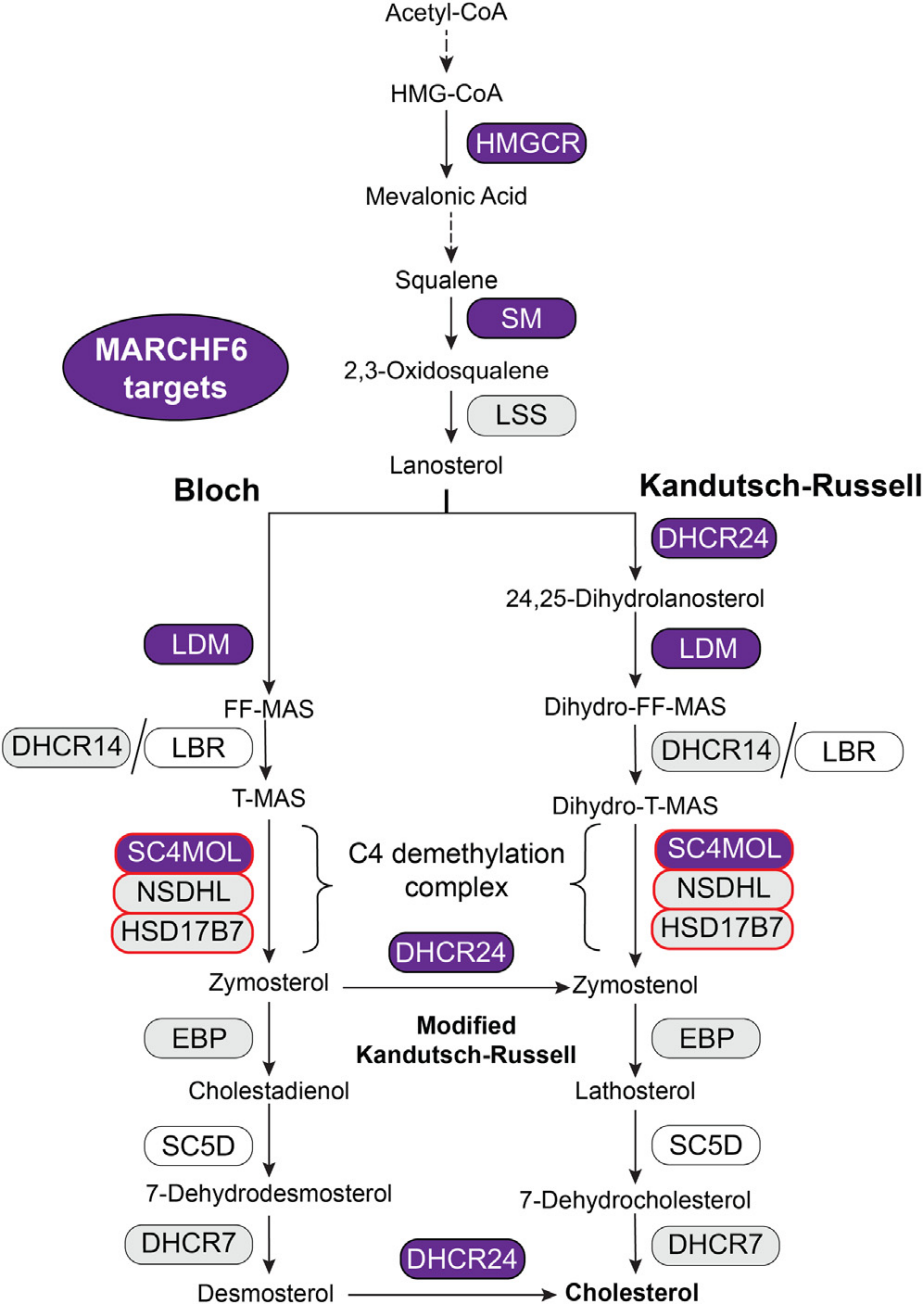
MARCHF6 targets in the cholesterol biosynthesis pathway. A simplified schematic of the cholesterol biosynthesis pathway showing all post-lanosterol enzymes and selected enzymes in the early pathway. Over 20 enzymes are required, where intermediates post lanosterol proceed via the Bloch pathway or Kandutsch-Russell pathway. DHCR24 can act on Bloch intermediates to shuttle them through the modified Kandutsch-Russell pathway. MARCHF6 targets (purple), tested non-MARCHF6 targets (gray), and untested enzymes (white) are indicated, with discoveries from this paper outlined in red.

MARCHF6 is an important regulator of cholesterol synthesis. Our previous work indicates it targets four cholesterol synthesis enzymes (HMGCR and SM (18), LDM, and DHCR24 (19)), and here we have found a fifth, SC4MOL (Fig. 6). However, MARCHF6 does not appear to target six others (lanosterol synthase and 3-β-hydroxysteroid-Δ^8^,Δ^7^-isomerase (19), DHCR14 (21), NSDHL and HSD17B7 (this work), and DHCR7 (20)) (Fig. 6). It remains to be determined whether MARCHF6 targets any of the remaining cholesterol synthesis enzymes. Interestingly, MARCHF6 tends to target resource-intensive enzymes; all five known targets use NADPH as a cofactor and three enzymes (SM, LDM, and SC4MOL) require oxygen.

Moreover, MARCHF6 itself is regulated by cholesterol; MARCHF6 undergoes autoubiquitination, and cholesterol prevents its autodegradation and stabilizes its levels, influencing levels of its substrates (35). MARCHF6 can therefore rapidly shut down the synthesis of cholesterol by targeting five resource-intensive steps. Curiously, cholesterol can interact with MARCHF6 and SC4MOL (52). Whether the cholesterol-mediated stabilization of MARCHF6 and cholesterol regulation of SC4MOL is the result of direct cholesterol binding is an outstanding question.

Intriguingly, MARCHF6 may be a link between ferroptosis and lipid metabolism by responding to NADPH and cholesterol levels. MARCHF6 was recently found to target the ferroptosis effectors p53 and ACSL4 for degradation (31). Significantly, MARCHF6 works as an NADPH sensor. NADPH binds to the C-terminal regulator region, which is involved in substrate discrimination and self-ubiquitination (31, 32). This interaction upregulates MARCHF6 E3 ligase activity and consequently downregulates ferroptosis (31). Cholesterol synthesis is an NADPH-demanding process, requiring 19 molecules of NADPH per cholesterol molecule, with the MARCHF6 substrates, HMGCR, SM, DHCR24, and LDM, and DHCR7 using between one and three molecules of NADPH each (53). Remarkably, of all the cholesterol biosynthesis enzymes, SC4MOL requires the most NADPH for catalysis, with six NADPH molecules used for complete C4-demethylation (Fig. 1). Given this C-terminal regulatory region binds NADPH, it is likely involved in SC4MOL degradation, although this awaits further investigation.

This is the first known instance of a single E3 ubiquitin ligase degrading multiple enzymes in the same biochemical pathway, in this case to coordinate a rapid shutdown of cholesterol synthesis. One other human E3 ubiquitin ligase, CTLH, targets two enzymes in one metabolic pathway but does not lead to their degradation (54). CTLH ubiquitinates two glycolytic enzymes, inhibiting their activity and reducing overall glycolytic flux without degrading the proteins. CTLH and MARCHF6 provide evidence for E3 ligases coordinating metabolic flux as needed. E3 ubiquitin ligases therefore provide a fresh perspective for understanding metabolic homeostasis.

MARCHF6 does not require lysines at SC4MOL’s cytosol-accessible C terminus to promote its degradation (Fig. 3). It may ubiquitinate lysines located elsewhere or select other residues. While canonical ubiquitination occurs on lysine residues, there is increasing evidence for non-lysine ubiquitination (55). In fact, we previously found MARCHF6 selects serine residues on SM for ubiquitination (56) and noncanonical ubiquitination may also be the case for SC4MOL. Further work with mutagenesis and/or mass spectrometry methods could determine MARCHF6 ubiquitinated residues on SC4MOL, as well as its other substrates.

In conclusion, SC4MOL is a highly regulated enzyme of the C4-demethylation complex in cholesterol biosynthesis. We identified SC4MOL as another MARCHF6 substrate, bringing the number of MARCHF6 cholesterogenic substrates to five. SC4MOL is turned over rapidly, transcriptionally and post translationally responsive to sterols, and its depletion results in a decrease in total cholesterol levels. Our work represents the first characterization of the regulation of SC4MOL and additionally implicates MARCHF6 as a crucial posttranslational regulator of cholesterol synthesis. Moreover, our work further reinforces the intriguing idea that a single E3 ubiquitin ligase can help coordinate metabolism by targeting multiple critical steps in a key biochemical pathway.

## Data availability

All data are contained within the article.

## Supplemental data

This article contains supplemental data (15, 18, 37, 57).

## Conflict of interest

The authors declare that they have no conflicts of interest with contents of this article.
