# Supplementary material for: Cholesterol synthesis enzyme SC4MOL is fine-tuned by sterols and targeted for degradation by the E3 ligase MARCHF6
Source: J Lipid Res. 2023 Mar 22;64(5):100362. doi: 10.1016/j.jlr.2023.100362 (PMC10176258; doi:10.1016/j.jlr.2023.100362)
Supplement: Supplemental Table S1 and Figures S1–S6 [file mmc1.docx]

**Supplemental Data**

**Supplementary Table 1**: Primers used for Nanobit cloning, SC4MOL site-directed mutagenesis and qRT-PCR. The His.NheI.cleave.N primer was used to clone SM and MARCHF6 N-BiT constructs. The His.EcoRI.cleave.C was used to clone MARCHF6 C-BiT constructs. The His.SacI.cleave.C was used to clone MARCHF6 N-BiT constructs.

| Primer | 5′ to 3′ Sequence | Reference |
| --- | --- | --- |
| SM N-BiT SacI | GGTGGTGAGCTCAGTGGACTTTTCTGGGCATTG | This study |
| SM C-BiT NheI | GAGTGAGCTAGCGATGTGGACTTTTCTGGGCATTGC | This study |
| MARCHF6 N-BiT EcoRI | GGTGGTGAATTCAGACACCGCGGAGGAAG | This study |
| MARCHF6 C-BiT NheI | GGTGGTGCTAGCATGGACACCGCGG | This study |
| His.NheI.cleave.N | TGAGCTAGCTAGCTCAATGGTGATGGTGATGATG | (15) |
| His.EcoRI.cleave.C | GGTGGTGAATTCCCATGGTGATGGTGATGATG | (15) |
| His.SacI.cleave.C | GAGTGAGAGCTCCATGGTGATGGTGATGATGACCGG | This study |
| SC4MOL K284R Forward | GTATAATGCCTATAATGAAAGGAGG | This study |
| SC4MOL K286-291R Reverse | TTCAGTCCTTCTCTCAAACCTCCTCCTCCTTTCATTATAGG | This study |
| PBGD Forward | GAGTGATTCGGGTGGGTACC | (57) |
| PBGD Reverse | GGCTCCGATGGTGAAGCC | (57) |
| MARCHF6 Forward | GAGGTACTTCGACCTGGTGTC | (18) |
| MARCHF6 Reverse | CACTGTAGAGCATGACATTG | (18) |
| NSDHL Forward | TGACCTCTGCAGCCGACAGG | (15) |
| NSDHL Reverse | CAGTTCCATTCTTGATATCGACGCCC | (15) |
| SC4MOL Forward | GAGTTTCAGGCTCCATTTGGAATGG | (15) |
| SC4MOL Reverse | GAAATCATGATGCCGAGAACCAGC | (15) |
| HSD17B7 Forward | GGATGTCAGCAACCTGCAGTCGG | (15) |
| HSD17B7 Reverse | CTCAAACACCTCCTGAAGTCCATCAGC | (15) |
| ERG28 Forward | TGGCTGGTTATGGTGTCCATC | (15) |
| ERG28 Reverse | ATGTCAATGGCACAGAGGCAG | (15) |


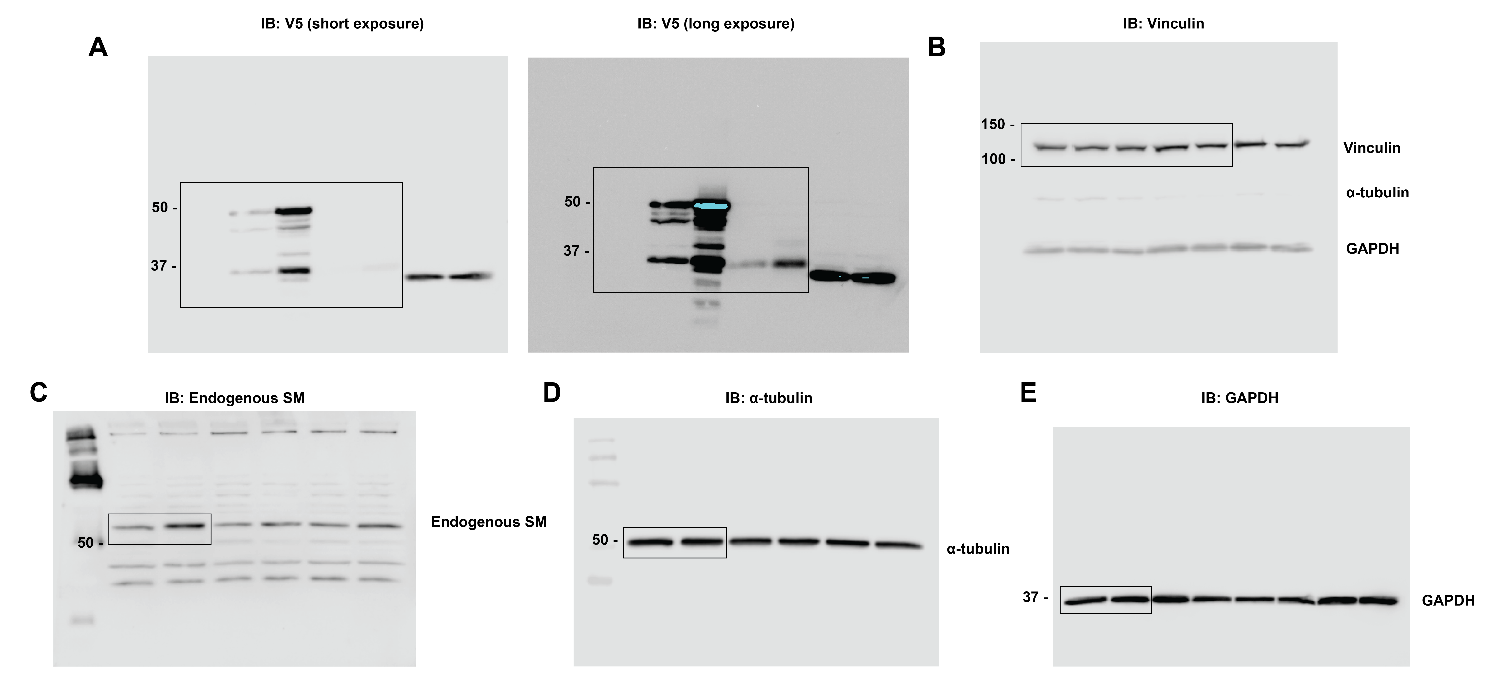


**Supplementary Figure 1: Full Western Blots of antibodies used.**

(A) V5, (B) Endogenous Vinculin, (C) Endogenous SM, (D) Endogenous α-tubulin and (E) Endogenous GAPDH blots shown in Figure 1 and supplementary Figure 3. Boxes indicate cropped image used in the final figures. Numbers indicate the kDa marker.


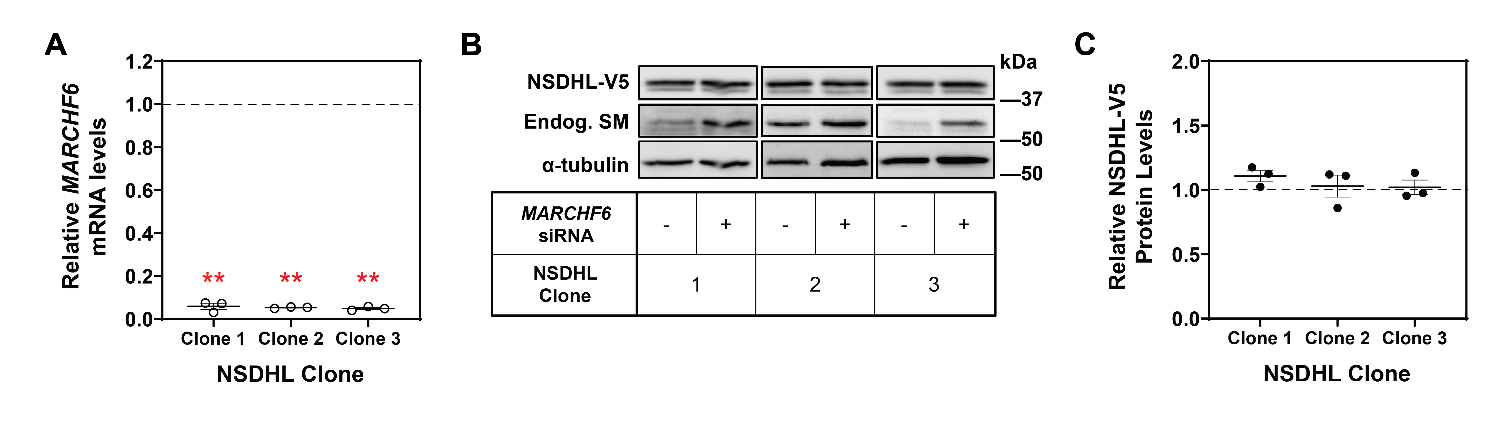


**Supplementary Figure 2: NSDHL is not a likely MARCHF6 target.**

(A-C) Three clones of CHO-NSDHL-V5 cells were transfected with 25 nM control (-) or *MARCHF6* (+) siRNA for 24 h. *MARCHF6* mRNA levels were measured using qRT-PCR and normalized to the housekeeping gene *PBGD* and to the control condition, which was set to 1 (represented by dashed line). (B) Protein levels were analysed by Western blotting with V5, endogenous SM and α-tubulin antibodies. (C) Relative protein levels were measured using ImageStudio Lite and normalized to the control condition which was set to 1 (represented by dashed line). Data presented as mean ± SEM from n = 3 independent experiments performed in triplicates (A) or n = 3 independent experiments (B), where **p <0.01. A paired student’s two-tailed *t*-test was conducted between control and *MARCHF6* siRNA knockdown.


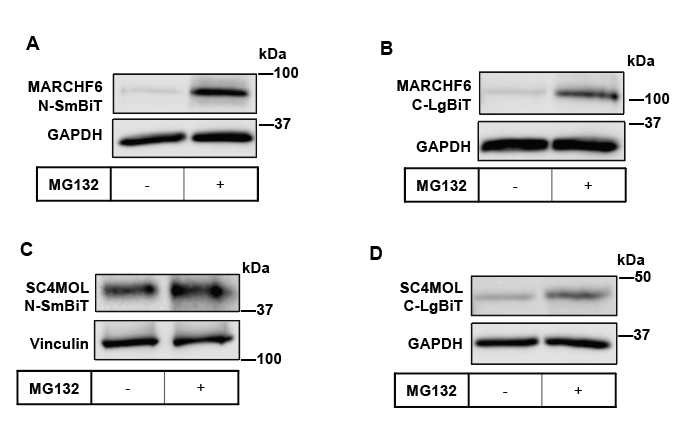


**Supplementary Figure 3: Protein levels of SC4MOL and MARCHF6 NanoBiT Constructs increase after MG132 treatment.**

HEK293T cells were seeded and transfected with NanoBiT constructs indicated for 24 h. Cells were treated with or without 5 µM MG132 for 16 before protein harvest. Protein levels were analysed by Western blotting with V5, endogenous SM, GAPDH and vinculin antibodies. Blots are representative of n = 1 experiment.


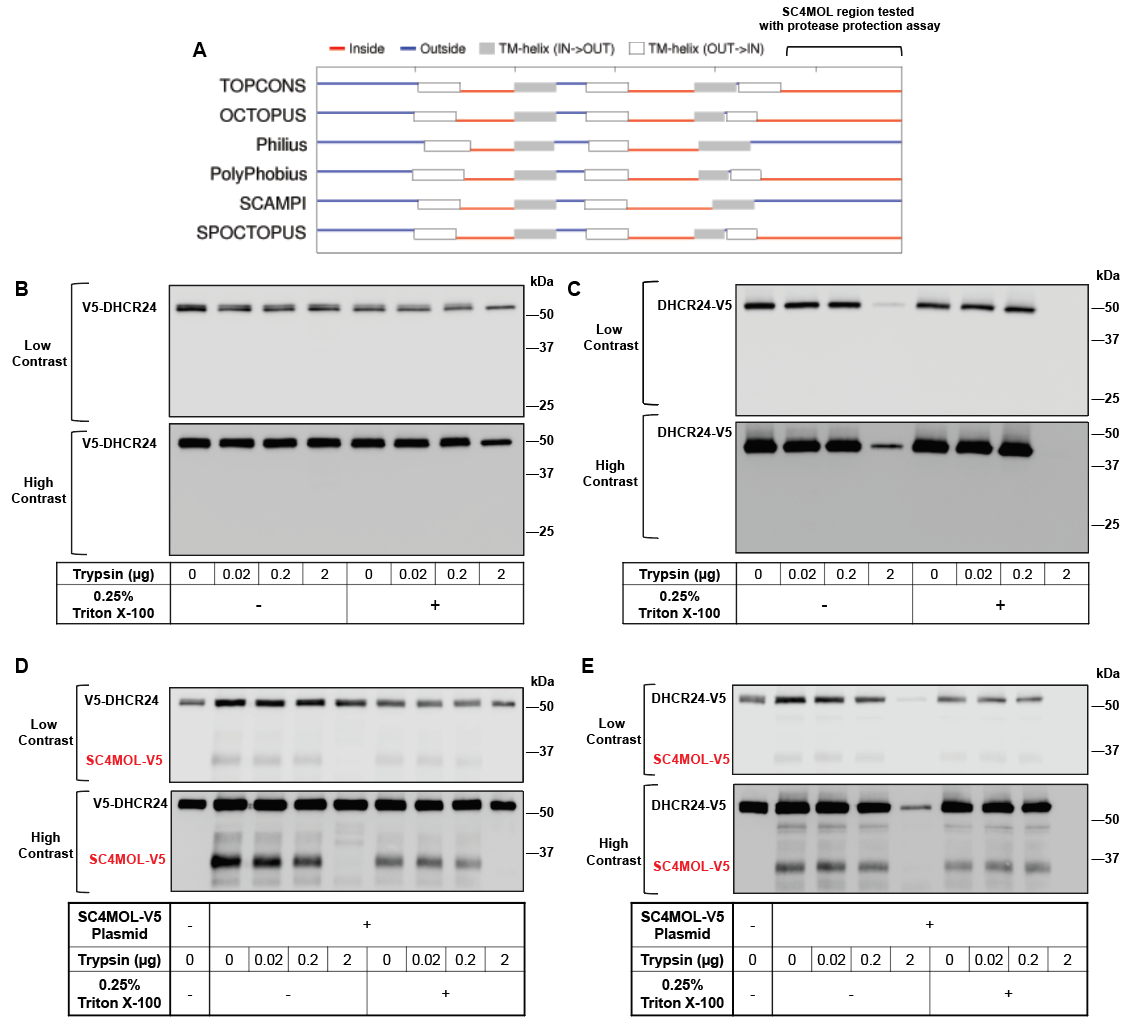


**Supplementary Figure 4**: Protease protection assay of SC4MOL-V5.

(A) Topology prediction of SC4MOL with TOPCONS (37). (B, C) CHO-7 cells were transfected with 4 µg of EV and (B) 1 µg of V5-DHCR24 or (C) DHCR24-V5 in a 10 cm dish for 24 h. (D, E) CHO-7 cells were co-transfected with 4 µg SC4MOL-V5 and 1 µg of (D) V5-DHCR24 or (E) DHCR24-V5 in a 10 cm dish for 24 hours. Cell lysate was fractionated, and membranes were isolated. Digest was performed with the indicated amount of trypsin in the presence or absence of Triton X-100. Membranes were separated by 10% SDS-PAGE. DHCR24 and SC4MOL proteins were immunoblotted with antibodies against the V5 epitope. Data representative of n = 2 experiments (B, C) or n = 3 experiments (D, E).

**
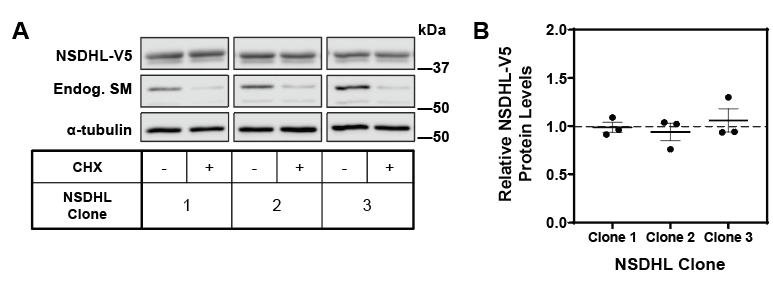
**

**Supplementary Figure 5: NSDHL is highly stable.**

(A and B) Three clones of CHO-NSDHL-V5 were treated with or without 10 μg/mL cycloheximide (CHX) for 8 h. (A) Protein levels were analysed by Western blotting with V5, endogenous SM and α-tubulin antibodies. (B) Protein levels relative to the control condition for each clone, set to 1 (represented by dashed line). Data presented as mean ± SEM from *n* = 3 independent experiments.


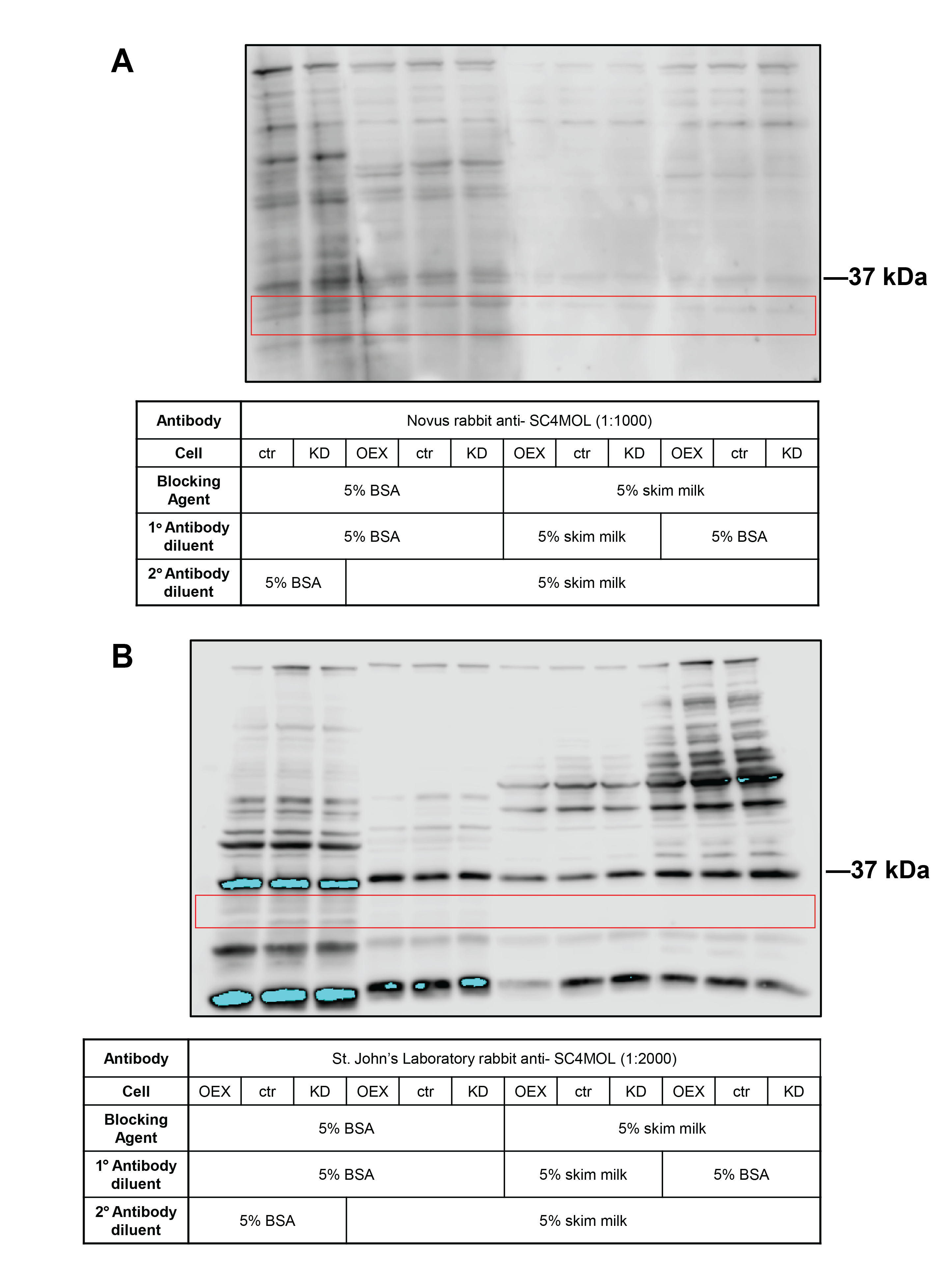


Supplementary Figure 6: Testing endogenous SC4MOL antibodies.

HEK293T cells were transfected with SC4MOL-V5 plasmid (OEX) or control (ctr) or *SC4MOL* siRNA (KD) for 24 h before protein harvest. Endogenous antibodies from Novus (A) and St. John’s Laboratory (B) were diluted according to manufacturer’s instructions and probed for 1 h at room temperature in diluents specified. Secondary rabbit antibody in 5% BSA in PBST or 5% skim milk in PBST were tested. Red box indicates expected position of endogenous SC4MOL.
